# Prevalence of lumbar spinal stenosis, using the diagnostic support tool, and correlated factors in Japan: a population-based study

**DOI:** 10.1007/s00776-013-0455-5

**Published:** 2013-08-21

**Authors:** Shoji Yabuki, Norio Fukumori, Misa Takegami, Yoshihiro Onishi, Koji Otani, Miho Sekiguchi, Takafumi Wakita, Shin-ichi Kikuchi, Shunichi Fukuhara, Shin-ichi Konno

**Affiliations:** 1 Department of Orthopedic Surgery, Fukushima Medical University School of Medicine, Fukushima, Fukushima 960-1295 Japan; 2 Department of Healthcare Epidemiology, Graduate School of Medicine and Public Health, Kyoto University, Kyoto, Japan; 3 Department of Preventive Medicine and Epidemiologic Informatics, National Cerebral and Cardiovascular Center, Osaka, Japan; 4 Institute for Health Outcomes and Process Evaluation Research (iHope International), Kyoto and Tokyo, Japan; 5 Faculty of Sociology, Kansai University, Osaka, Japan

## Abstract

**Background:**

Few studies have examined the prevalence of lumbar spinal stenosis (LSS) in the general population. The purposes of this study were to estimate the prevalence of LSS and to investigate correlated factors for LSS in Japan.

**Methods:**

A questionnaire survey was performed on 4,400 subjects selected from residents aged 40–79 years in Japan by stratified two-stage random sampling in 2010. The question items consisted of lower-limb symptoms suggestive of LSS, the diagnostic support tool for LSS (LSS-DST), demographic and lifestyle characteristics, comorbidities, the Japanese Perceived Stress Scale (JPSS), and the Mental Health Index 5 (MHI-5). Using the LSS-DST, the presence of LSS was predicted to estimate the prevalence of LSS. Logistic regression analysis was performed to examine the relationship between LSS and correlated factors.

**Results:**

Questionnaires were obtained from 2,666 subjects (60.6 %), consisting of 1,264 males (47.4 %). The mean (standard deviation) age was 60.0 (10.9) years. According to the LSS-DST, 153 subjects were regarded as having LSS. The prevalence was estimated to be 5.7 %. When standardizing this value with the age distribution of the Japanese population, it was estimated that 3,650,000 Japanese subjects aged 40–79 years might have LSS using the LSS-DST. Prevalence increased with age and was particularly high in subjects aged 70–79 years, irrespective of gender. As correlated factors, an advanced age (60 years or older), diabetes mellitus, urological disorders, and osteoarthritis/fracture as comorbidities, and depressive symptoms, were associated with LSS.

**Conclusions:**

This study elucidated the prevalence of LSS and factors associated with LSS in Japan. This is the first report describing the estimated prevalence of LSS and associated factors using a strictly sampled representative population.

## Introduction

Lumbar spinal stenosis (LSS) is defined as a syndrome in which narrowing of the spinal canal and intervertebral foramen, which are nervous routes, related to degeneration of the lumbar intervertebral disks and/or joints, causes specific symptoms of the lumbar region and lower limbs [1–3]. Patients with this disease complain of numbness and pain in the lumbar and gluteal regions, and intermittent claudication leads to gait disorder in some cases [2, 4]. Although stenosis of the spinal canal is associated with organic abnormalities, for example spondylolysis and spondylolisthesis in some patients, aging-related degeneration of the vertebral bodies and/or intervertebral disks may be etiologically involved in most patients [2]. Therefore, this disease has been regarded as common in elderly subjects.

However, few large-scale epidemiological surveys involving the general population have been conducted to investigate the prevalence of LSS, possibly because it was difficult to apply subjects’ symptom and CT/MRI finding-based clinical LSS diagnosis [5] to a large-scale survey. Therefore, previous epidemiological studies had limitations such as a small sample size or study sample restricted to those with abnormal imaging findings [6, 7]. Furthermore, the prevalence of LSS varied among the studies, probably because of a lack of standardized methods and criteria for diagnosis. Therefore, it was difficult to interpret and compare the survey results.

In epidemiological studies on LSS in Japan, the study samples were also limited to a population in a single city or patients who consulted a hospital with symptoms [8–10]. Konno et al. [11] developed a diagnostic support tool for LSS (LSS-DST) based on a self-administered questionnaire regarding patients’ symptoms for subjects with lower-limb symptoms suggestive of LSS. This facilitated large-scale questionnaire surveys on the presence or absence of LSS involving the general population.

As risk factors associated with LSS, aging, gender, and body mass index (BMI) have been reported [12, 13]. However, these studies also involved only patients with abnormal imaging findings of the vertebrae. Therefore, to date, no study has investigated risk factors for LSS in the general population.

The purpose of this study was to clarify the prevalence of LSS-associated lower-limb symptoms, estimate the prevalence of LSS, and investigate factors associated with LSS, using a cross-sectional survey involving a representative sample selected from Japanese residents.

## Materials and methods

### Subjects

This study was the JOA-Subsidized Science Project Research 2009 and conducted on commission from the Japanese Orthopaedic Association. The protocol was approved by the ethics review board of Fukushima Medical University.

The study population consisted of all residents aged 40–79 years old in Japan who were able to respond to a questionnaire. Sampling was performed by stratified two-stage random sampling. Briefly, as a first step, the nation was divided into 43 layers through a 9-area and 5-city scale, and 200 points were selected on the basis of the population of each regional block and/or city-scale-classified layer. As a second step, 22 subjects per point were randomly selected from “the Basic Resident Registration” to obtain 4,400 representative samples. We conducted a cross-sectional survey involving questionnaire placement along with visits to the subjects’ homes. The survey period was from November to December 2010.

### Measurements

To examine the presence or absence of lower-limb symptoms associated with LSS, the following question was initially asked: Have you experienced pain, numbness, or flushes of the lower limbs (gluteal region, femoral region, and lower thigh) within the last 1 month?. Simultaneously, a figure was attached so that respondents could accurately understand the sites of the gluteal region, femoral region, and lower thigh. In subjects who reported the presence of lower-limb symptoms, we predicted the presence or absence of LSS by use of the LSS-DST. The LSS-DST consists of 10 question items and has a sensitivity and specificity of 84 and 78 %, respectively [11]. The items of a self-administered history questionnaire as the LSS-DST are shown in Table 1. Each item of the LSS-DST for diagnosis of LSS required a response either of “1 = yes” or “0 = no”. A total score of 4 on Q1–Q4 or a score >1 on Q1–Q4 and >2 on Q5–Q10 indicated the presence of LSS.

**Table 1:** The items of a self-administered, self-reported history questionnaire (SSHQ) as a diagnostic support tool for LSS

	Item
Q1	Numbness and/or pain in the thighs down to the calves and shins
Q2	Numbness and/or pain increase in intensity after walking for a while, but are relieved by taking a rest
Q3	Standing for a while brings on numbness and/or pain in the thighs down to the calves and shins
Q4	Numbness and/or pain are reduced by bending forward
Q5	Numbness is present in both legs
Q6	Numbness is present in the soles of both feet
Q7	Numbness arises around the buttocks
Q8	Numbness is present, but pain is absent
Q9	A burning sensation arises around the buttocks
Q10	Walking nearly causes urination

The questionnaire included Mental Health Index 5 (MHI-5), which was measured using 5 items in the subscale of the Medical Outcomes Study 36-Item Short Form Health Survey (SF-36) [14–16]. Subjects with an MHI-5 score of 51 or lower, 52–59, and 60–67 were regarded as having severe, moderate, and mild depressive symptoms, respectively. To measure stress in daily living, the Japanese Perceived Stress Scale (JPSS) [17–19] was used. This is a scale that measures the level of perceived daily stress, but not the stress from a life event. Using the total JPSS score, the level of perceived stress was classified into 3 groups: weak (<18), moderate (≥18, <23), and strong (≥23). The questionnaire also included questions about age, gender, comorbidities, educational background, household income, marital status, smoking status, and alcohol consumption.

### Statistical analysis

The prevalence of lower-limb symptoms associated with LSS is presented. On the basis of the presence or absence of LSS predicted using the LSS-DST, we estimated the prevalence of LSS in the Japanese population. In addition, on the basis of the population with regard to age in Japan in 2010 (Statistics Bureau, Ministry of Internal Affairs and Communications) [20], the data were standardized using the direct method to estimate the number of patients with LSS in Japan.

Multivariate logistic regression analysis was performed to examine risk factors for LSS. In this model, age, gender, comorbidities, educational background, household income, marital status, smoking, alcohol consumption, perceived daily stress, and depressive symptoms were used as explanatory variables. For statistical analysis, Stata SE version 12 software (Stata, USA) was used.

## Results

**Fig. 1:**
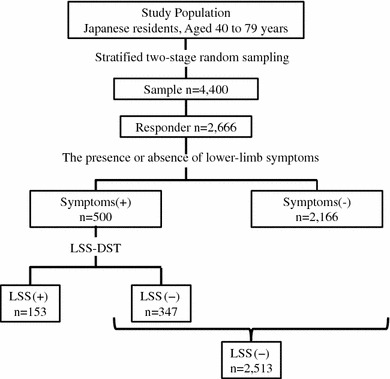
Flow chart of the population-based study for prevalence of lumbar spinal stenosis (LSS)

Of the 4,400 subjects, questionnaires were obtained from 2,666 (response 60.6 %) (Fig. 1). The mean age (standard deviation) of the respondents was 60.0 (10.9) years. The proportion of males was 47.4 %. The respondents’ characteristics are shown in Table 2.

**Table 2:** Characteristics of subjects (*n* = 2,666)

	*n* (%)		*n* (%)
Age (mean ± SD = 60.0 ± 10.9)		Marital status	
40–49	572 (21.5)	Unmarried	180 (6.8)
50–59	694 (26.0)	Married	2,056 (77.1)
60–69	789 (29.6)	Separated/divorced/bereaved	363 (13.6)
70–79	611 (22.9)	Smoking status	
Sex		Never	1,790 (67.1)
Male	1,264 (47.4)	Past	180 (6.8)
Female	1,402 (52.6)	Current	574 (21.5)
BMI (kg/m^2^)		Alcohol drinking	
<18	129 (4.8)	Hardly ever or never	1,441 (54.1)
≥18, <22	988 (37.1)	Sometimes	469 (17.6)
≥22, <25	916 (34.4)	Almost every day	683 (25.6)
≥25, <30	458 (17.2)	Exercise habit	
≥30	175 (6.6)	No	1,300 (48.8)
Number of comorbidities		Yes	1,253 (47.0)
0	984 (36.9)	Occupation	
1	713 (26.7)	No	1,536 (57.6)
2≤	969 (36.4)	Yes	1,067 (40.0)
Educational level		Perceived Stress (JPSS)	
Elementary-high school	1,191 (44.7)	Low (<18)	815 (30.6)
Professional school, Junior college	327 (12.3)	Moderate (18–22)	756 (28.4)
University or above	360 (13.5)	Severe (≥23)	1,095 (41.1)
Household income (JPY)		Depressive symptoms (MHI-5)	
<3,000,000	645 (24.2)	None (≥68)	1,635 (61.3)
3,000,000≤, <5,000,000	665 (24.9)	Low (60–67)	424 (15.9)
5,000,000≤, <7,000,000	461 (17.3)	Moderate (52–59)	168 (6.3)
7,000,000≤	633 (23.7)	Severe (<52)	380 (14.3)

*SD* Standard deviation, *BMI* body mass index, *JPY* Japanese yen, *JPS* Japanese Perceived Stress Scale, *MHI-5* Mental Health Inventory-5

Of the respondents, 500 (18.8 %) had complained of LSS-associated symptoms of the lower limbs (pain, numbness, flushes) within 1 month before the survey (Fig. 1). The prevalence of lower-limb symptoms increased with age irrespective of gender, reaching a maximum at 70 years of age.

**Fig. 2:**
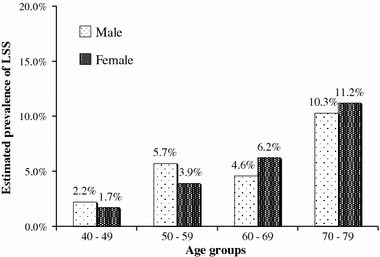
Estimated prevalence of lumbar spinal stenosis (LSS) with regard to age

Of the respondents, 153 were regarded as having LSS according to the LSS-DST (crude prevalence of 5.7 %). With regard to age (Fig. 2), the prevalence of LSS was estimated to be 1.9 % in respondents aged 40–49 years, 4.8 % in those aged 50–59 years, 5.5 % in those aged 60–69 years, and 10.8 % in those aged 70–79 years, showing an increase with age. With regard to gender, the prevalence of LSS in males and females of all ages was 5.7 and 5.8 %, respectively, showing no difference. However, in males and females aged 70–79 years, the prevalence was 10.3 and 11.2 %, respectively, which was slightly higher in females. Standardizing with the age distribution of the Japanese population, the age-adjusted prevalence of LSS using the LSS-DST was estimated to be 5.7 %, and the number of patients with LSS was estimated to be 3,650,000, among Japanese aged 40–79 years. The prevalence of LSS estimated with regard to residential area and urban scale is shown in Table 3. With regard to residential area, the prevalence in the Kanto (6.4 %) and Kinki (7.6 %) regions, in which the Japanese population is concentrated, was estimated to be high. However, with regard to urban scale, there were no notable trends in the estimated prevalence of LSS.

**Table 3:** The prevalence of lumbar spinal stenosis (LSS) in all subjects by local areas and the population size of the cities

	No. of subjects	LSS(+)
		*n * (%)
Local areas of Japan		
Hokkaido	138	7 (5.1)
Tohoku	241	9 (3.7)
Kanto	849	54 (6.4)
Hokuriku	146	6 (4.1)
Tokai	337	14 (4.2)
Kinki	381	29 (7.6)
Chugoku	163	6 (3.7)
Shikoku	74	6 (8.1)
Kyusyu	337	22 (6.5)
Population size of the cities		
19 Largest cities^a^	625	34 (5.4)
≥200,000	627	40 (6.4)
≥100,000	483	27 (5.6)
<100,000	648	37 (5.7)
Rural areas	283	15 (5.3)

^a^The 19 largest cities are Sapporo, Sendai, Saitama, Chiba, Tokyo, Yokohama, Kawasaki, Niigata, Shizuoka, Hamamatsu, Nagoya, Kyoto, Osaka, Sakai, Kobe, Okayama, Hiroshima, Kitakyusyu, Fukuoka

Among the respondents, we compared their characteristics between subjects who were regarded as having LSS by use of the LSS-DST and those who were not regarded as having LSS (non-LSS) (Table 4). In the subjects with LSS, the mean age was more advanced, and the number of comorbidities was greater. Among the comorbidities examined, hypertension, diabetes, cardiovascular diseases (myocardial infarction, stroke, and angina pectoris), ophthalmic disorder, gastrointestinal disorder, urological disorder, osteoarthritis/fracture, neurological disorder, and osteoporosis were concomitantly present in larger proportions of subjects with LSS than in those without (non-LSS).

**Table 4:** Comparison of characteristics between subjects with and without lumbar spinal stenosis (LSS)

	LSS(−)	LSS(+)	*P* value
	*n *(%)	*n *(%)	
Age (mean ± SD = 60.0 ± 10.9)			<0.001
40–49	561 (22.3)	11 (7.2)	
50–59	661 (26.3)	33 (21.6)	
60–69	746 (29.7)	43 (28.1)	
70–79	545 (21.7)	66 (43.1)	
Sex			0.934
Male	1,192 (47.4)	72 (47.1)	
Female	1,321 (52.6)	81 (52.9)	
BMI (kg/m^2^)			0.113
<18	124 (4.9)	5 (3.3)	
≥18, <22	940 (37.4)	48 (31.4)	
≥22, <25	865 (34.4)	51 (33.3)	
≥25, <30	420 (16.7)	38 (24.8)	
≥30	164 (6.5)	11 (7.2)	
Educational level			0.006
Elementary-high School	1,100 (43.8)	91 (59.5)	
Professional school, Junior college	314 (56.3)	13 (8.5)	
University or above	346 (70.0)	14 (9.2)	
Household income (JPY)			0.004
<3,000,000	593 (23.6)	52 (34.0)	
3,000,000≤, <5,000,000	624 (24.8)	41 (26.8)	
5,000,000≤, <7,000,000	446 (17.8)	15 (9.8)	
7,000,000≤	603 (24.0)	30 (19.6)	
Marital status			0.520
Unmarried	173 (6.9)	7 (4.6)	
Married	1,935 (77.0)	121 (79.1)	
Separated/divorced/bereaved	340 (13.5)	23 (15.0)	
Smoking status			0.786
Never	1,690 (67.3)	100 (65.4)	
Past	168 (6.7)	12 (7.8)	
Current	541 (21.5)	33 (21.6)	
Alcohol drinking			0.788
Hardly ever or never	1,363 (54.2)	78 (51.0)	
Sometimes	445 (17.7)	24 (15.7)	
Almost every day	642 (25.6)	41 (26.8)	
Exercise habit			0.122
No	1,236 (49.2)	64 (41.8)	
Yes	1,173 (46.7)	80 (52.3)	
Occupation			0.003
No	1,466 (58.3)	70 (45.8)	
Yes	988 (39.3)	79 (51.6)	
Perceived stress (JPSS)			<0.001
Low (<18)	787 (31.3)	28 (18.3)	
Moderate (18–22)	720 (28.7)	36 (23.5)	
Severe (≥23)	1,006 (40.0)	89 (58.2)	
Depressive symptoms (MHI-5)			<0.001
None (≥68)	1,574 (62.6)	61 (39.9)	
Low (60–67)	394 (15.7)	30 (19.6)	
Moderate (52–59)	157 (6.3)	11 (7.2)	
Severe (<52)	333 (13.3)	47 (30.7)	
Number of comorbidities			<0.001
0	954 (38.0)	30 (19.6)	
1	685 (27.3)	31 (20.3)	
2≤	874 (34.8)	92 (60.1)	
Comorbidities			
Hypertension	658 (26.2)	66 (43.1)	<0.001
Diabetes mellitus	199 (7.9)	30 (19.6)	<0.001
Hyperlipidemia	388 (15.4)	25 (16.3)	0.731
Cardiovascular disease	161 (6.4)	24 (15.7)	<0.001
Ophthalmic disorder	255 (10.2)	34 (22.2)	<0.001
Respiratory disorder	124 (4.9)	12 (7.8)	0.127
Gastrointestinal disorder	267 (10.6)	25 (16.3)	0.033
Hematological disorder	146 (5.8)	10 (6.5)	0.721
Kidney disease	63 (2.5)	8 (5.2)	0.062
Urological disorder	116 (4.6)	19 (12.4)	<0.001
Osteoarthritis/fracture	172 (6.8)	36 (23.5)	<0.001
Rheumatoid arthritis	49 (2.0)	3 (2.0)	1.000
Dermatological disorder	109 (4.3)	8 (5.2)	0.543
Neurological disorder	7 (0.3)	3 (2.0)	0.016
Psychiatric disorder	69 (2.8)	6 (3.9)	0.443
Endocrine disorder	83 (3.3)	3 (2.0)	0.483
Pancreatic disorder	26 (1.0)	3 (2.0)	0.230
Malignant disease	79 (3.1)	7 (4.6)	0.340
Gynecological disorder	108 (4.3)	11 (7.2)	0.104
Osteoporosis	95 (3.8)	17 (11.1)	<0.001
Others	98 (3.9)	12 (7.8)	0.032

*SD* Standard deviation, *BMI* body mass index, *JPY* Japanese yen, *JPS* Japanese Perceived Stress Scale, *MHI-5* Mental Health Inventory-5

The associations of the subjects’ characteristics and mental factors with the presence of LSS are shown in Table 5. The adjusted odds ratio of LSS increased with age. In subjects aged 70–79 years, it was 5.38 times higher than that in those aged 40–49 years (95 % confidence interval (95 %CI) 2.03–14.21, respectively). There were no significant associations with gender or BMI. Concerning the number of comorbidities, the crude odds ratio was 3.38 in subjects with 2 or more comorbidities. However, the adjusted odds ratio of this association was not significant. With regard to individual comorbidities, there was an increase in the adjusted odds ratio in the presence of urologic disorder (2.17, 95 %CI 1.10–4.29), diabetes mellitus (2.05; 95 % CI: 1.14–3.67), and osteoarthritis/fracture (2.71, 95 % CI 1.53–4.82). In subjects with severe depressive symptoms as a mental factor, the adjusted odds ratio was 3.55 (95 % CI 1.97–6.40).

**Table 5:** Associations of individual factors and comorbidities with prevalence of lumbar spinal stenosis (LSS)

	Crude analysis^a^			Adjusted analysis^a^		
	OR	95 % CI	*P* value	AOR	95 % CI	*P* value
Age (vs. 40–49)						
50–59	2.55	(1.28–5.08)	0.008	2.30	(0.93–5.65)	0.070
60–69	2.94	(1.50–5.75)	0.002	2.50	(1.00–6.21)	0.049
70–79	6.18	(3.23–11.81)	<0.001	5.38	(2.03–14.21)	0.001
Sex (vs. Male)						
Female	1.02	(0.73–1.41)	0.928	0.93	(0.57–1.53)	0.784
BMI (vs. ≥22, <25)						
<18	0.68	(0.27–1.75)	0.427	0.47	(0.10–2.16)	0.333
≥18, <22	0.87	(0.58–1.30)	0.486	1.40	(0.81–2.40)	0.228
≥25, <30	1.53	(0.99–2.37)	0.054	1.89	(1.08–3.32)	0.027
≥30	1.14	(0.58–2.23)	0.707	0.78	(0.27–2.26)	0.642
Educational level (vs. elementary-high school)						
Professional school, junior college	0.50	(0.28–0.91)	0.022	0.75	(0.37–1.50)	0.415
University or above	0.49	(0.28–0.87)	0.015	0.74	(0.38–1.47)	0.392
Household income (JPY) (vs. <3,000,000)						
3,000,000≤, <5,000,000	0.75	(0.49–1.15)	0.183	0.85	(0.49–1.46)	0.548
5,000,000≤, <7,000,000	0.38	(0.21–0.69)	0.001	0.58	(0.27–1.25)	0.165
7,000,000≤	0.57	(0.36–0.90)	0.017	0.88	(0.46–1.68)	0.709
Occupation (vs. no)						
Yes	1.674	(1.20–2.33)	0.002	0.80	(0.47–1.36)	0.409
Perceived stress (JPSS) (vs. low)						
Moderate (18–22)	1.41	(0.85–2.33)	0.186	1.47	(0.79–2.75)	0.229
Severe (≥23)	2.49	(1.61–3.84)	<0.001	1.50	(0.81–2.77)	0.197
Depressive symptoms (MHI-5) (vs. none)						
Low (60–67)	1.96	(1.25–3.08)	0.003	1.86	(1.02–3.38)	0.043
Moderate (52–59)	1.81	(0.93–3.51)	0.080	1.34	(0.53–3.37)	0.538
Severe (<52)	3.64	(2.45–5.42)	<0.001	3.55	(1.97–6.40)	<0.001
Comorbidities						
Hypertension	2.14	(1.53–2.98)	<0.001	1.21	(0.75–1.96)	0.428
Diabetes mellitus	2.84	(1.86–4.34)	<0.001	2.05	(1.14–3.67)	0.017
Cardiovascular disorder	2.72	(1.71–4.32)	<0.001	1.50	(0.77–2.94)	0.234
Ophthalmic disorder	2.53	(1.69–3.78)	<0.001	0.97	(0.53–1.78)	0.932
Gastrointestinal disorder	1.64	(1.05–2.57)	0.029	1.23	(0.70–2.15)	0.478
Urological disorder	2.93	(1.75–4.91)	<0.001	2.17	(1.10–4.29)	0.026
Osteoarthritis, Fracture	4.19	(2.79–6.27)	<0.001	2.71	(1.53–4.82)	0.001
Neurological disorder	7.16	(1.83–27.97)	0.005	7.87	(1.20–51.58)	0.031
Osteoporosis	3.18	(1.85–5.48)	<0.001	0.89	(0.38–2.08)	0.792

^a^The crude analysis used single-variate logistic regressions whereas the adjusted analysis used multivariate logistic regression including all explanatory variables

*OR* Odds ratio, *AOR* adjusted odds ratio, *BMI* body mass index, *JPY* Japanese yen, *JPSS* Japanese perceived stress test, *MHI-5* Mental Health Inventory-5

## Discussion

This study showed that in Japanese aged 40 to 79 years the prevalence of LSS-associated lower-limb symptoms was 18.8 %; the prevalence of LSS was estimated to be 5.7 % using the diagnostic support tool; and the number of patients with LSS was estimated to be 3,650,000.

Yamazaki [8] reported that the prevalence of LSS was 12.5 %, but their survey was limited to a single city, and the mean age was 67.9 years, more advanced than for the population in this study. This may have contributed to the higher reported prevalence. In this study, a sample representing the Japanese population was selected by a stratified two-stage random sampling method. The response was 60.6 %. Furthermore, there were no differences in gender or age distribution between responders and non-responders. Therefore, the results of this study can be generalized to Japanese aged 40–79 years. In previous epidemiological studies, prevalence of LSS of 1.7–22.5 % was reported. Kalichman et al. reported prevalence of 22.5 % using data from the Framingham Heart Study. They diagnosed LSS by using multidetector computed tomography (CT) to assess coronary and aortic calcification, irrespective of the presence or absence of clinical symptoms in the lower limbs. In this study a subject could be diagnosed as having LSS only when he/she complained symptoms in the lower limbs. Therefore, we might have detected clinically more relevant patients with LSS.

The estimated prevalence of LSS increased with age irrespective of gender. In particular, among elderly subjects, the prevalence in females was slightly higher than in males. This was consistent with the results of a previous study [12]. One reason to explain the slightly higher prevalence in females could be that aging-related degeneration of the vertebral bodies [2], which is the major etiology of LSS, may be closely related to osteoporosis in elderly females. However, in this study, the presence or absence of osteoporosis was not measured objectively, and its effect could not be clarified.

This study showed that factors associated with the presence of LSS were age, some comorbidities, and severe depressive symptoms. We found no significant associations between BMI and the presence of LSS in this study. In contrast Venkatesan et al. [21] reported high BMI as a risk factor for cauda equina syndrome. The population of their study was young (mean age of 39 years) and could include patients with diseases other than LSS, for example lumbar disc herniation, which may cause the association between BMI and cauda equina syndrome. With regard to individual comorbidities, the presence of urological disorders and osteoarthritis/fracture was associated with the presence of LSS. The prevalence of these conditions increased with age. Concerning urological disorders, in addition to this, the presence of neurogenic bladder, as an LSS-related cauda equina symptom, may be associated. Aortic calcification was reported to correlate with degeneration of the intervertebral discs and spine [22, 23]. Diabetes mellitus, one of the causes of LSS, can also increase the risk of aortic calcification [24], and this may be an explanation of the association with the presence of LSS. Concerning neurological disorders, the questionnaire asked neurological disorders as “neurological disorders such as epilepsy” and did not discern actual diagnoses. Therefore the neurological disorders may have contained peripheral nerve disorders which could cause symptoms resembling those by LSS. This is one explanation for the association between neurological disorders and LSS in this study. No study has reported an association between the presence of LSS and depression. In this study, the adjusted odds ratio of severe depressive symptoms was as high as 3.39. Sinikallio et al. [25] reported the prevalence of depression of 20 % among the patients with LSS which was higher than that in general population. Guilfoyle et al. [15] reported an association between depressive symptoms and low back pain. These findings suggest the presence of low back pain, including LSS, may be associated with the presence of depressive symptoms. In this study, we elucidated the association between the level of depressive symptoms and the presence of LSS in the representative sample of the Japanese population, which was allowed by using the self-reported diagnostic support tool for LSS. Concerning the association between the presence of LSS and perceived stress in daily living, we found no significant association in the multivariate analysis. A previous study has shown a low correlation between perceived stress in daily living measured by JPSS and depressive symptoms measured by MHI-5, suggesting that those two concepts are different each other [19, 26]. The MHI-5 contains items gauging psychological factors such as anxiety and stress, whereas JPSS contains physiological items in addition to psychological ones. Such differences may have caused the inconsistent results of associations of JPSS and MHI-5 with the presence of LSS shown in this study.

This study has some limitations. First, a questionnaire survey was used, so neither lower-limb symptoms nor LSS could be assessed objectively. For this reason, the accuracy of LSS diagnosis may not be particularly high. For example, some patients having related symptoms and a disease other than LSS, for example lumbar disc herniation, might be misclassified as LSS. However, we used a diagnostic support tool to predict LSS based on patients’ symptoms for the two reasons:

1. It is impossible to diagnose LSS by use of diagnostic imaging procedures, for example MRI, alone. Even when imaging shows abnormal findings, clinical symptoms can be absent in some patients [26, 27].
2. No standardized diagnostic criteria for comprehensive evaluation based on imaging findings and clinical symptoms have been established.

The sensitivity of the LSS-DST was 84 % and it is useful for predicting LSS in clinical settings. The sensitivity and specificity of the LSS-DST may differ depending on study settings. Because there are fewer subjects with typical symptoms of LSS or with suggestive symptoms in general populations than in clinical settings, the sensitivity could be lower and the specificity could be higher. The prevalence estimated in this study may differ from the true value and possibly have been underestimated. In this study, LSS was predicted only in subjects who complained of LSS-related lower-limb symptoms; therefore, clinically relevant LSS patients should have been selected. Second, this was a cross-sectional study, and we cannot indicate the causal relationship with risk factors for the development of LSS. In the future, a longitudinal study should be conducted to elucidate the causal relationship of LSS with depressive symptoms, as was suggested in this study.

In conclusion, we estimated the prevalence of LSS-related lower-limb symptoms and LSS in a representative sample of the Japanese population. The involvement of mental factors in LSS was suggested, which should be further examined in a future longitudinal study. The prevalence of LSS will increase with further advances in the rapid aging of society. Therefore, the prevalence of LSS and associated factors for LSS revealed in this study may provide information important for the future development of medical policies.
